# Nano Cobalt-Loaded Porous Carbon Derived from Waste Plastic for Efficient Persulfate Activation and Tetracycline Degradation

**DOI:** 10.3390/nano15050371

**Published:** 2025-02-27

**Authors:** Yueyue Luo, Xiuxiu Zhang, Yu Zhang, Jianchao Wang, Chongqing Wang

**Affiliations:** 1 Zhongyuan Critical Metal Laboratory, School of Chemical Engineering, Zhengzhou University, Zhengzhou 450001, China; luoyueyuezzu@163.com (Y.L.); zxx19960120@163.com (X.Z.); zhangyu001019@163.com (Y.Z.); 2 State Key Laboratory of Nutrient Use and Management, College of Resources and Environmental Sciences, National Academy of Agriculture Green Development, Key Laboratory of Plant–Soil Interactions (Ministry of Education), China Agricultural University, Beijing 100193, China

**Keywords:** advanced oxidation process, antibiotics, cobalt-loaded porous carbon, waste plastic

## Abstract

The excessive utilization and emission of waste plastics have caused serious damage to the environment, and it is of great significance to explore high-value utilization methods for these waste plastics. To address this challenge, functional nano cobalt-loaded porous carbon materials (CoPC) with excellent antibiotic wastewater removal properties were prepared by one-step pyrolysis using waste PET plastics as a carbon source, a process described in this paper. Characterization revealed that the obtained CoPC-2 catalysts had a high degree of defects, a large specific surface area (343.41 m^2^/g), and an abundant pore structure. Degradation results displayed that CoPC-2 removed 87.93% of 20 mg/L tetracycline with a reaction rate constant of 0.0668 min^−1^. Moreover, CoPC-2 exhibited excellent degradation performance for tetracycline over a wide range of pH levels (4–10) and in coexistence with multiple inorganic anions. Electron paramagnetic resonance and radical quenching experiments revealed that radicals (·OH, and SO_4_·^−^) and non-radicals (^1^O_2_) pathway participated in tetracycline degradation, with the non-radical pathway being dominant. This study not only offers promising prospects for resource utilization of waste plastics, but also provides novel approaches for the design of functional nanomaterials for antibiotic wastewater treatment.

## 1. Introduction

Since the 20th century, antibiotics have been extensively utilized in various fields such as agriculture, food processing, cultivation, medicine, and animal husbandry [1]. As the demand for antibiotics has witnessed a dramatic surge, a gradual increase in the frequency of antibiotics being detected in the environment has been observed [2]. As an emerging pollutant, antibiotics such as levofloxacin (LVF), tetracycline (TC), ciprofloxacin (CIP), ofloxacin (OFX), norfloxacin (NOR), and sulfamethoxazole (SMX), after accumulating in the environment for a long time, will undermine aquatic ecosystems and have an impact on both the dynamic balance of biological communities and human health [3,4,5,6,7,8,9]. Currently, the treatment approaches for antibiotic wastewater primarily include adsorption, biological remedies, membrane separation, and advanced oxidation processes (AOPs) [10,11,12]. However, compared with other methods that have various shortcomings, such as adsorbent selectivity and regeneration problems, long treatment periods, and sensitivity to environmental conditions, sulfate radical-based AOPs exhibit remarkable advantages, such as high degradation efficiency, potent oxidizing capacity (SO_4_·^−^, 2.5~3.1 V), a broadly applicable pH range, straightforward application, and no generation of secondary pollution, which make them an important means for removing difficult-to-degrade pollutants in aquatic environments [13,14,15,16,17,18,19]. Unfortunately, persulfates, including peroxymonosulfate (PMS) and peroxydisulfate (PDS), are too relatively stable to release free radicals for contaminant degradation in their normal state, and thus require activation.

Transition metals, heat, UV light, and carbon materials are the methods typically used to activate persulfates [20,21,22]. Among these methods, transition metals, such as cobalt (Co), iron (Fe), and nickel (Ni), but especially Co, are ideal candidates for persulfate activators because of their high activation efficiency without additional energy input [1,23,24]. However, transition metals are highly pH-dependent, i.e., they tend to form hydroxide precipitates at alkaline pH values, leading to separation and recovery difficulties. In addition, the activation of transition metal catalysts will be obscured due to agglomeration phenomena, leading to a reduction in catalytic efficiency [25,26]. Therefore, to expand their applicable range and improve the recovery efficiency of transition metal catalysts, it is essential to select suitable carriers for transition metals. Carbon materials’ good electrical conductivity, diverse pore structure, large specific surface area, and relative stability render them the best choices for carriers [27,28,29,30]. For instance, a cobalt cross-linked ordered mesoporous carbon material (OMC-Co-T800) was synthesized by evaporation-induced self-assembly, which was able to remove up to 99% of SMX (10 mg/L) within 30 min [2]. Furthermore, a cobalt-embedded carbon aerogel (Co-CA-900) was prepared from lotus root starch as a raw material, and the Co-CA-900/PMS system was able to remove 95.3% of CBZ in 20 min [31]. By impregnation and one-step rapid pyrolysis, Liu’s team embedded Co nanoparticles into biochar prepared from rose petals (Co@RBC800). In the Co@RBC800/PMS system, LVF could achieve 100% degradation at 15 min with strong stability and negligible Co ion leaching [32]. Therefore, loading transition metals onto carbon materials to improve the degradation efficiency and stability of catalysts is a feasible option. However, the source of the core carbon source of the carbon material is an issue that needs to be considered.

Plastics, synthetic or semi-synthetic materials boasting traits such as being lightweight, highly plastic, highly chemically stable, and low cost, have found extensive applications in various industries, including packaging, construction, medicine, and transport [33,34]. With the ever-growing demand for plastics, global plastic production has already exceeded 8300 million metric tons since 2017 [35]. With the consumption of plastic products increasing, the amount of waste plastics has also progressively grown. As of 2015, 6.3 billion tons of plastic waste were reportedly generated globally, of which only 9% was recycled, and 12 billion tons of waste plastic is expected to be generated by 2050 [36,37]. The long-term accumulation of waste plastics in the natural environment not only results in a tremendous waste of resources, but also destroys the ecological environment, such as affecting soil structure, impeding the growth of crops, polluting bodies of water, and endangering marine life [38,39]. Therefore, low-cost and efficient resource utilization of waste plastics is extremely necessary. Given the high carbon content of waste plastics, carbonization has emerged as a novel approach to convert various discarded plastics into value-added carbon nanomaterials and carbon-based composites [34,40]. The resulting carbonaceous materials, including porous carbon, activated carbon, carbon nanotubes, graphene, carbon fibers, etc., have promising applications in the fields of batteries [35], supercapacitors [41], pollutant removal [42,43], solar energy evaporation [44], and CO_2_ capture [45,46].

Therefore, to realize the high-value utilization of waste plastics and efficient degradation of antibiotics in wastewater, this study innovatively selected waste PET plastics as a carbon source to prepare functional nano cobalt-loaded porous carbon materials (CoPC) with special properties by one-step pyrolysis. By controlling the loading amount of Co ions, the structure of the CoPC catalysts was regulated to improve the utilization of active centers and the catalytic activity of the catalysts. The structural properties of CoPC and the performance of activated PMS for the removal of TC wastewater were examined. Furthermore, the active sites and catalytic mechanisms of CoPC composites for TC degradation were investigated. This not only has a positive effect on solving the pollution problems caused by antibiotics in wastewater, but also provides a new strategy for advanced PMS oxidation technology and high-value utilization of waste plastics.

## 2. Experimental Section

### 2.1. Materials and Characterizations

The waste plastic was obtained from waste beverage bottles, whose main component is polyethylene terephthalate (PET). The chemicals used in the experiment were all analytically pure, and their specific information is shown in Text S1 of the Supporting Information. In addition, a series of characterizations for the prepared catalysts were carried out, and the instrumental information is shown in Text S2 of the Supporting Information.

### 2.2. Preparation Method of CoPC Catalysts

**Figure 1:**
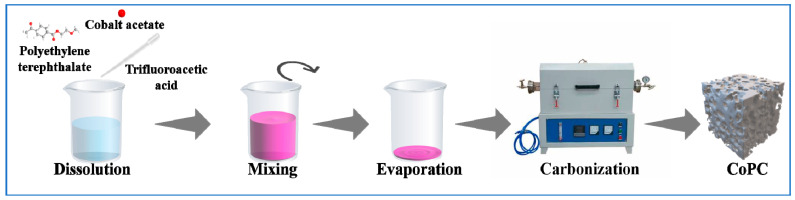
Preparation diagram of the CoPC catalyst.

Figure 1 shows the preparation of the CoPC catalyst. Discarded PET plastic bottles were cut into 1 cm × 1 cm solid sheets. A total of 2.0 g of PET and different ratios of (CH_3_COO)_2_Co·4H_2_O (CA) were added to 12 mL of CF_3_COOF for 2 h with stirring (PET:CA = 6:1, 4:1, 2:1). Subsequently, the resulting mixture was stirred at 80 °C until the solvent was completely evaporated to form a well-mixed PET/CA mixture. The obtained PET/CA was placed in a tube under an N_2_ atmosphere at an elevated temperature which increase by a rate of 5 °C/min up to 800 °C and carbonized for 1 h to obtain nano cobalt-doped porous carbon materials (CoPC). The carbonized samples of PET:CA = 6:1, 4:1, and 2:1 were denoted as CoPC-1, CoPC-2, and CoPC-3, respectively. Meanwhile, the same carbonization process was carried out on the pure PET plastic without Co loading, which was named PET-800.

### 2.3. Catalytic Degradation Test

TC was chosen as the target contaminant. The TC degradation process is specifically described in Text S3 of the Supporting Information. The TC degradation rate was calculated from the absorbance of TC at 354 nm (Equation (1)). Each set of experiments was repeated at least twice.

(1) $$\eta =\frac{C_{0}-C_{t}}{C_{0}}\times 100\%$$

where: C_0_ and C_t_ are the initial and final TC concentration (mg/L), respectively, and η is the TC degradation rate (%).

## 3. Results and Discussion

### 3.1. Characterization of the Nanocatalyst

**Figure 2:**
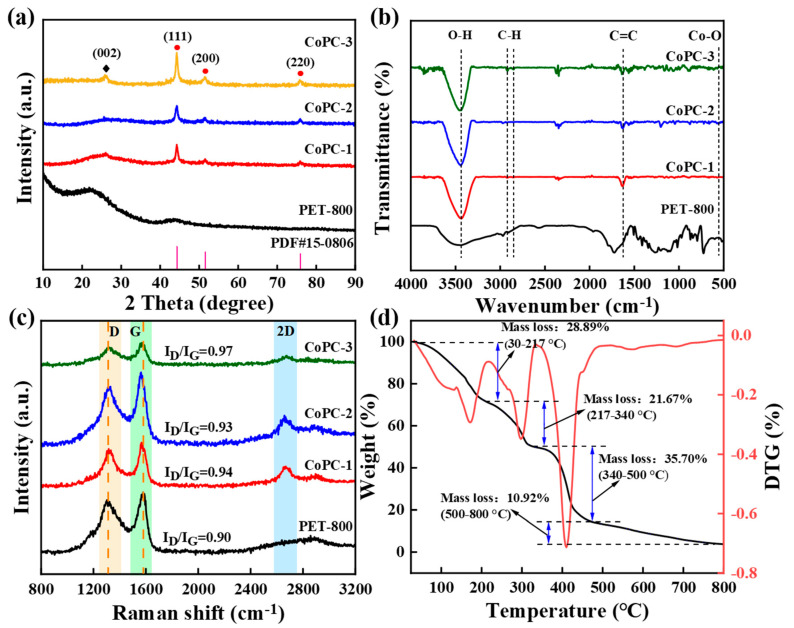
(**a**) XRD, (**b**) FT-IR, and (**c**) Raman spectra of PET-800 and CoPC catalysts; (**d**) Thermogravimetric curve of the CoPC-2 catalyst precursor.

The XRD pattern in Figure 2a displayed that the peak around 26.1° for PET-800 and CoPC was assigned to the carbon (002) crystal plane. For the CoPC catalyst, the characteristic peaks at 2θ = 44.2°, 51.6°, and 75.8° were the (111), (200), and (220) crystalline planes of Co^0^, respectively (PDF#15-0806) [23]. This suggested that the cobalt element was present in the CoPC catalyst as Co^0^ nanoparticles after calcination. Furthermore, the intensity of the Co^0^ nanoparticles diffraction peaks gradually increased with increases in cobalt loading. FT-IR spectra displayed that some basic characteristic peaks corresponding to the carbon material could be observed (Figure 2b). Stretching vibrations of the O-H group and the C=C band appeared around 3440 and 1625 cm^−1^, respectively [47]. The expansion vibration of the -CH_2_ group was responsible for the small absorption peaks at 2921 and 2859 cm^−1^ [48]. The spectral band at 560 cm^−1^ was attributed to the Co-O bond, proving the successful loading of cobalt elements in the CoPC composites [31].

Raman spectroscopy was employed to investigate the structural differences between PET-800 and CoPC composites with different Co loading amounts. The D band and G band were observed in the Raman spectra of all samples (Figure 2c) [49]. Compared with PET-800, CoPC catalysts showed a red-shifted D peak and blue-shifted G peak, which was probably due to Co decoration induced changes in the electronic structure of the porous carbon materials [50]. The I_D_/I_G_ intensity ratios of CoPC-1, CoPC-2, and CoPC-3 were 0.94, 0.93, and 0.97, respectively, which were all higher than that of 0.90 for PET-800, suggesting that Co loading increased the degree of defects and disorder in the carbon material. The I_D_/I_G_ values of the CoPC composites showed a phenomenon of decreasing and then increasing with the increase in cobalt loading. Research reported that a moderate amount of cobalt could promote the graphitizing of carbon carriers through catalysis, while excessive cobalt would lead to a large number of defects on the carbon-based material surface, thus reducing the degree of graphitization [13]. In addition, CoPC-2 had the strongest 2D peak, indicating fewer structural defects and a higher crystalline quality of CoPC-2, which corresponded to the change in I_D_/I_G_ values [47].

The thermo-gravimetric curve in Figure 2d showed that the weight loss of the precursor of the CoPC-2 catalyst was divided into four major processes. In the range of 30 °C–217 °C, the weight loss of 28.89% was mainly attributed to the escape of physically adsorbed water and residual trifluoroacetic acid solvent from the catalyst [43]. The mass loss in the second stage (217 °C–340 °C) was about 21.67%, which was related to the decomposition of cobalt acetate and organic matter in the PET polymer [13]. The PET polymer backbone was pyrolyzed between 340 °C–500 °C, resulting in the largest weight loss (35.70%) [51,52]. Simultaneously, cobalt acetate is further decomposed into cobalt oxides [53]. The last stage of weight loss may be related to the escape of CO and CO_2_ fractions due to the pyrolytic decomposition of carbonyl and carboxylic ester-containing functional groups in the PET matrix [54]. The results displayed that PET plastic was converted into a carbon skeleton and cobalt acetate decomposed into cobalt monomers or cobalt oxides as the temperature increased, proving the feasibility of preparing cobalt/carbon composites by carbonizing plastic.

**Figure 3:**
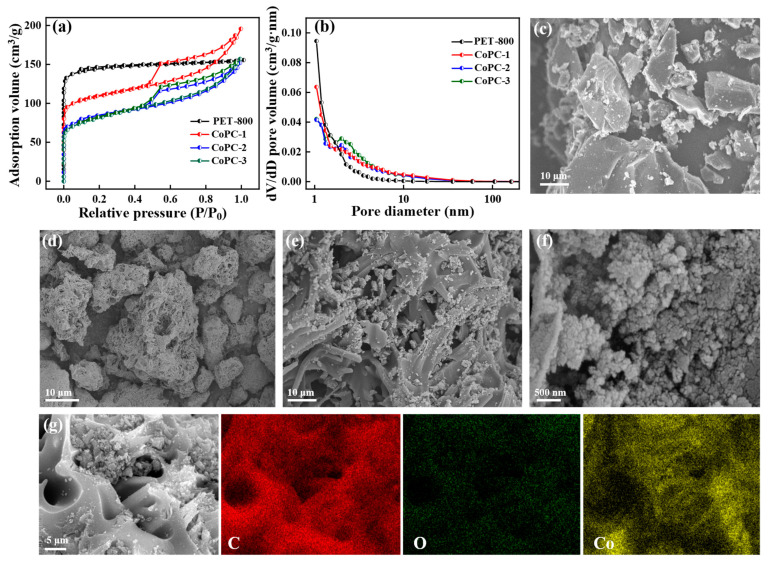
(**a**) N_2_ adsorption–desorption isotherms and (**b**) pore size distribution curves of PET-800 and CoPC catalyst; SEM images of (**c**) PET-800 and (**d**–**f**) CoPC-2 catalyst; (**g**) EDS mapping of CoPC-2.

The results of the N_2_ adsorption–desorption isotherms in Figure 3a and Table S1 exhibit the fact that the specific surface area (SSA) of PET-800 was 438.64 m^2^/g, which was significantly larger than that of CoPC-1 (262.45 m^2^/g), CoPC-2 (343.41 m^2^/g), and CoPC-3 (266.52 m^2^/g). This phenomenon was presumed to be due to the Co loading forming a coating or deposit on the carbon material surface, resulting in the original pores being covered or blocked [48,55]. This speculation was supported by the SEM images of PET-800 and CoPC-2. PET-800 presented an irregular shape with a smooth surface (Figure 3c), whereas the surface of CoPC-2 presented a honeycomb porous structure, with obvious Co nanoparticle stacking and pore clogging, and the size of the stacked Co nanoparticles was about 100–200 nm (Figure 3d–f). By EDS elemental mapping analysis (Figure 3g), it was found that Co nanoparticles accumulated on the surface and in the pores of the carbon carriers. Moreover, both CoPC materials presented IV isotherms and their average pore sizes exceeded 2 nm, indicating that the CoPC composites possessed mesoporous structures (Figure 3b). The total pore volume of CoPC-2 was 0.2976 cm^3^/g, which was significantly higher than that of CoPC-1 (0.2337 cm^3^/g) and CoPC-3 (0.2409 cm^3^/g), indicating that CoPC-2 could provide more opportunities for TC to react with active sites and improve its catalytic efficiency [56].

### 3.2. Catalytic Performance of CoPC Catalyst

**Figure 4:**
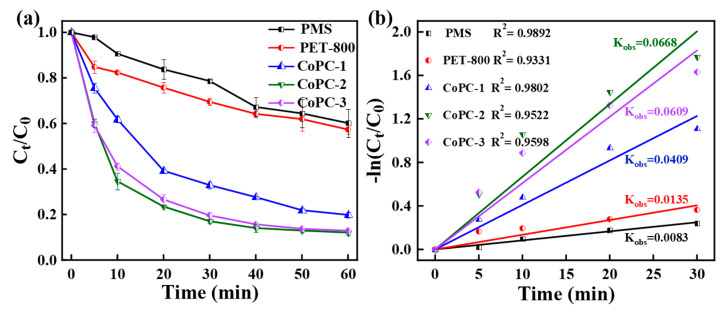
(**a**) Degradation performance of TC and (**b**) Linear fitting of pseudo-first-order kinetics of PMS, PET-800, and CoPC composites.

To evaluate the activity of CoPC catalysts with different Co loadings, the prepared catalysts were used for the activation of PMS to degrade TC (Figure 4). The TC removal by PET-800, CoPC-1, CoPC-2, and CoPC-3 catalysts in the absence of PMS was found to be 6.60%, 7.66%, 6.02%, and 4.28%, respectively, implying that the adsorption of TC by the catalysts was negligible (Figure S1). Moreover, about 39.84% of TC was removed when PMS alone was present, indicating the limited ability of PMS to degrade TC [1,31]. Meanwhile, in the PET-800/PMS system, the TC removal was only increased by about 3%, indicating that the PET-800 catalyst without Co nanoparticles loading had weak catalytic activity for PMS. Compared with the PET-800 catalyst, the CoPC-1, CoPC-2, and CoPC-3 catalysts showed a greater improvement in TC degradation within 60 min, with degradation rates of 80.15%, 87.93%, and 87.12%, respectively, which was higher than that of most of Co-based catalysts (Table S2) [1,2,5,7,8,9,32,48,57]. Fitting of the reaction rate constants (k_obs_) revealed that the k_obs_ of CoPC-2 (0.0668 min^−1^) was higher than that of CoPC-3 (0.0609 min^−1^), which could be attributed to the clogging of the catalyst’s pore structure due to excessive Co nanoparticle loading, leading to the shielding of the active groups exposed on the surface from interacting with PMS [2,13]. Therefore, considering the reaction rate and cost, the CoPC-2 catalyst was selected for an investigation of degradation factors and catalytic degradation mechanism.

**Figure 5:**
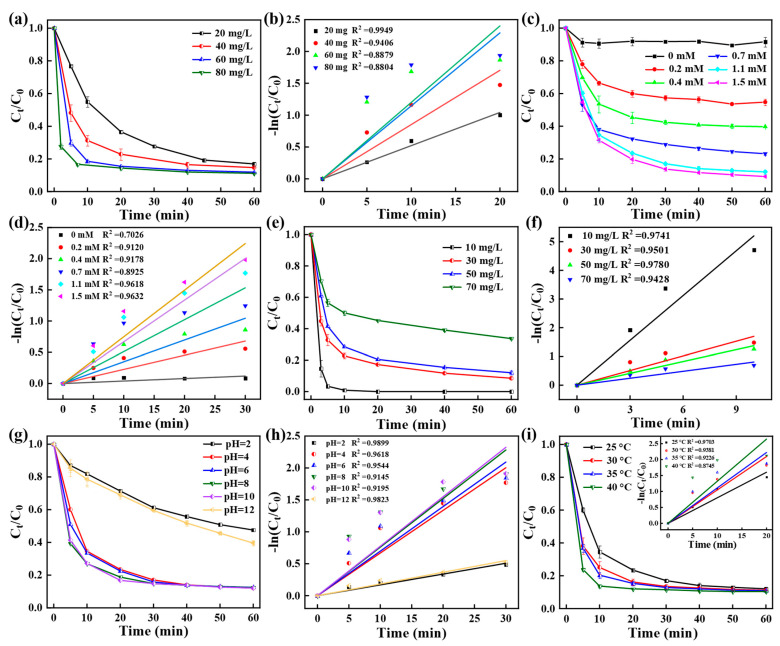
Effects of (**a**) catalyst dosage, (**c**) PMS dosage, (**e**) TC concentration, and (**g**) solution pH on TC degradation by CoPC-2; Linear fitting of the pseudo-first-order kinetic equation of (**b**) catalyst dosage, (**d**) PMS dosage, (**f**) TC concentration, and (**h**) solution pH; (**i**) Effects of solution temperature on TC degradation and linear fitting of the pseudo-first-order kinetic equation.

Factors such as the dosage of the CoPC-2 catalyst and PMS, the TC concentration, solution pH, and reaction temperature were examined to optimize the TC degradation conditions. As displayed in Figure 5a,b, an increasing trend in the TC degradation rate was found when the amount of CoPC-2 was increased from 20 to 60 mg/L, which can probably be explained by the fact that more active sites were provided to activate PMS, thus increasing the TC degradation rate [1]. However, when the dosage was further increased to 80 mg/L, the k_obs_ value only increased from 0.11456 to 0.12002 min^−1^ (Table S3). The slow increase in kinetic constants may be related to the limitations of the PMS dosage. Therefore, the effects of PMS dosage on TC removal efficiency were investigated in the following process.

The degradation performance of TC displayed an increase from 6.02% to 87.93% when the PMS dosage increased from 0 to 1.1 mM (Figure 5c), and the k_obs_ increased from 0.0039 to 0.0668 min^−1^ (Figure 5d and Table S4), probably because the high concentration of PMS provided more free radical species, which facilitated the degradation process. In contrast to the above, the improvement in TC removal was limited when the amount of PMS reached 1.5 mM, which may be ascribed to the bursting influence of SO_4_·^−^ as a result of an excessive amount of PMS [4,31]. Therefore, 1.1 mM PMS was selected as the optimal dosage. Subsequently, the influence of the TC concentration on TC removal was further investigated. Figure 5e,f shows that the degradation efficiency and k_obs_ tended to decrease with the increase of the initial TC concentration. For 10 mg/L TC, CoPC-2/PMS could achieve 100% degradation within 10 min, and the k_obs_ was as high as 0.4656 min^−1^ (Table S5). For an initial concentration of 70 mg/L, TC degradation performance reduced to 66.28% within 60 min, which was caused by the limited amount of reactive oxygen species (ROSs). Moreover, the excess TC would adsorb on the catalyst surface and compete with PMS for the active sites on the catalyst, leading to a decrease in the activity of the CoPC-2 catalyst [55]. Therefore, the initial concentration of TC should not be too high.

The pH value is an essential parameter that affects the activation of PMS. In the range of pH = 2~12, the degradation efficiency of the CoPC-2/PMS system on TC was investigated (Figure 5g,h). The degradation of TC was basically unaffected in the pH range of 4~10, maintaining a high degradation performance. Under excessively acidic (pH = 2) or alkaline (pH = 12) conditions, TC degradation was inhibited to varying degrees. At pH = 2, the rate of removal of TC was 52.43%, which was attributed to the binding of hydrogen ions to HSO_5_^−^ in the strongly acidic environment limiting the activation process [13,47]. For pH = 12, the TC degradation efficiency was also suppressed to 60.39% (Table S6). The reason may be that the more oxidizing SO_4_·^−^ is easily converted to the less oxidizing ∙OH under alkaline conditions, leading to a decrease in catalytic activity [58]. Moreover, the reaction temperature significantly affected the degradation of TC in the CoPC-2/PMS system. Figure 5i and Table S7 demonstrated that the k_obs_ increased from 0.0802 to 0.1321 min^−1^ as the temperature increased from 25 to 40 °C, indicating that the reaction process was thermally absorptive and that higher temperatures were favorable for the reaction to proceed. According to the Arrhenius equation, it was calculated that the CoPC-2/PMS system had an activation energy of 24.18 kJ/mol (Figure S2), which was lower than that of S-Co-MOF@400 (48.56 kJ/mol) [58] and S_0.3_-Co@P_2_C (41.51 kJ/mol) [13].

**Figure 6:**
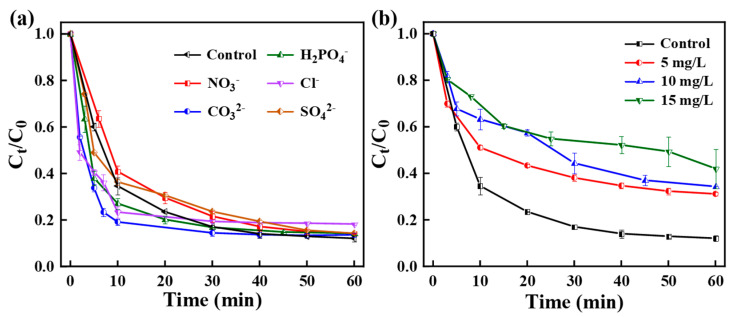
Influences of (**a**) inorganic anions and (**b**) humic acid on TC degradation.

The presence of inorganic anions also affects the degradation behavior of the target pollutants through various pathways during the actual wastewater treatment process. Figure 6a examined the influences of SO_4_^2−^, Cl^−^, NO_3_^−^, CO_3_^2−^, and H_2_PO_4_^−^ (10 mM) on TC removal efficiency. The co-existence of NO_3_^−^, SO_4_^2−^, and H_2_PO_4_^−^ had a weak influence on the degradation of TC in the CoPC-2/PMS system, indicating that the CoPC composites are highly resistant to ionic interference. Cl^−^ exhibited a weak inhibitory effect on TC, which was ascribed to the production of less active chloride ions by Cl^−^ as a free radical scavenger [2]. In addition, CO_3_^2−^ was found to promote the degradation of TC in a short period. The reason was probably that CO_3_^2−^ could react with ∙OH to produce carbonate radicals (CO_3_**^·^**^−^), which was also involved in the degradation of TC [59]. Moreover, humic acid (HA) was taken as a representative of natural organic matter to investigate its effect on TC removal. Figure 6b demonstrated that in the presence of 5, 10, and 15 mg/L of HA, the decomposition rate of TC decreased to 68.84%, 65.63%, and 57.6%, respectively. The gradual decrease in the catalytic activity of CoPC-2 could be attributed to the competitive reaction between HA and TC on the active radicals. In addition, the high concentration of HA was able to burst ROSs, resulting in the reduction of ROSs [2].

### 3.3. Discussion of Degradation Mechanisms

**Figure 7:**
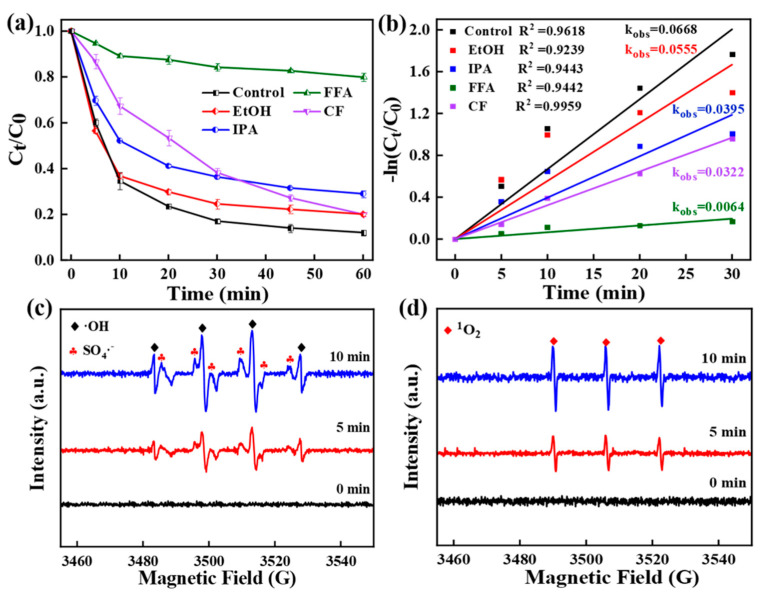
(**a**) Influence of radical scavengers on TC degradation; (**b**) Linear fitting of the pseudo-first-order kinetic equation; EPR spectra of (**c**) ·OH and SO_4_·^−^, and (**d**) ^1^O_2_ in the CoPC-2/PMS system.

The reactive species related to the activated persulfate decomposition process of metal/carbon composites have been reported to include radicals (·OH, SO_4_·^−^, O_2_·^−^) and non-radicals (^1^O_2_). Therefore, the reactive groups of CoPC-2 which activated PMS degradation of TC were identified by quenching experiments and EPR tests (Figure 7a). Depending on the reaction rate of the capture agent with the radicals, ethanol (EtOH) was used to capture SO_4_·^−^ (1.6 × 10^7^ M^−1^·s^−1^) and ·OH (k = 1.9 × 10^9^ M^−1^·s^−1^), and isopropanol (IPA) and chloroform (CF) were utilized to capture the ·OH (k = 3.8–7.6 × 10^8^ M^−1^·s^−1^) and O_2_·^−^ (k = 3.8–7.6 × 10^8^ M^−1^·s^−1^) [59]. The TC degradation efficiency was reduced by 8.02%, 16.91%, and 6.45%, while the k_obs_ decreased to 0.0555, 0.0395, and 0.0322 min^−1^ when 10 mM EtOH, IPA, and CF were added to the reaction system, suggesting that the SO_4_·^−^, ·OH, and O_2_·^−^ dominated radical pathways contribute weakly to TC removal in the CoPC-2/PMS system. Moreover, the generation pathway of O_2_·^−^ was explored. The influence of dissolved oxygen (O_2_) on TC degradation was investigated by using N_2_ to evacuate O_2_ from the reaction system (Figure S3). Results displayed that the presence of O_2_ had essentially no effect on TC degradation, suggesting that O_2_·^−^ production was not dependent on O_2_ [10]. Therefore, it was speculated that non-radical degradation pathways also existed in the reaction system. Furfuryl alcohol (FFA) was used to capture ^1^O_2_ [1]. After the addition of FAA to the system, the removal of TC decreased dramatically from 87.93% to 20.08%, and the k_obs_ was suppressed to 0.0064 min^−1^, suggesting that ^1^O_2_ displayed a dominant role (Figure 7b). Radical quenching experiments demonstrated the existence of ·OH, SO_4_·^−^, and O_2_·^−^ radical pathways as well as ^1^O_2_-dominated non-radical pathways in the CoPC-2/PMS system, and the non-radical pathways were dominant.

**Figure 8:**
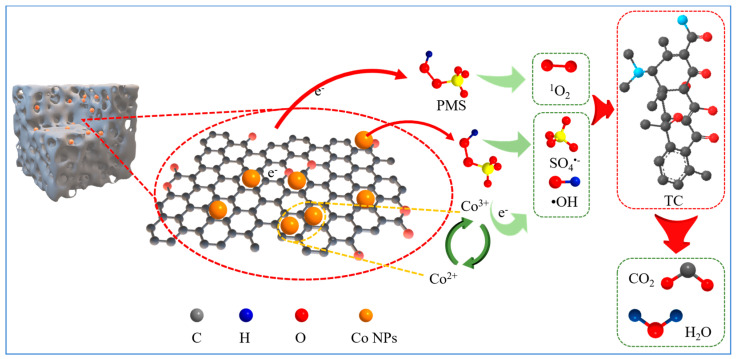
Degradation mechanism of TC in the CoPC-2/PMS system.

To further validate the results of the capture experiments, the reactive groups of the CoPC-2/PMS reaction system were examined using EPR. No signal peak was observed in the presence of PMS alone (0 min). With the addition of the CoPC-2 catalyst to the reaction system, signal peaks for ·OH (intensity ratio 1:1:1:1:1:1), SO_4_·^−^ (intensity ratio 1:2:2:1), and ^1^O_2_ (intensity ratio 1:1:1) were detected (Figure 7c,d). Importantly, the signal intensities gradually increased with the prolongation of the reaction time, proving the existence of ·OH, SO_4_·^−^, and ^1^O_2_ in the reaction system. Therefore, a possible catalytic mechanism for TC degradation was proposed (Figure 8). During TC degradation, both SO_4_·^−^, ·OH, and ^1^O_2_ generated by CoPC-2-activated PMS contributed. Studies have reported that the redox reaction between cobalt oxide (Co^2+^ and Co^3+^) and PMS on the surface of cobalt-based catalysts produces ROSs [47,60]. For the production of ^1^O_2_, it was demonstrated that oxygen-containing functional groups can form surface-reactive complexes with PMS, which facilitated the electron transfer pathway and ^1^O_2_ generation [2,13,61]. These activators mineralize the TC molecules into small molecule intermediates as well as CO_2_ and H_2_O.

## 4. Conclusions

In brief, nano cobalt-loaded porous carbon materials (CoPC) with excellent catalytic performance were successfully prepared by using waste plastic as a carbon source. Owing to the catalytic effect of cobalt ions during the pyrolysis process, the prepared CoPC catalysts possessed a high degree of defects and an abundant pore structure. Degradation results showed that CoPC-2 could remove up to 87.93% of 20 mg/L TC when the PMS concentration was 1.1 mM and the catalyst dosage was 20 mg/L. In addition, CoPC-2 maintained a high degradation performance for TC in the pH range of 4–10, and a high range of applicability in the presence of inorganic anions and low concentrations of humic acid. Investigation of the catalytic degradation mechanism revealed that both radical (·OH and SO_4_·^−^) and non-radical (^1^O_2_) pathways are involved in the degradation of TC, with the non-radical pathway predominating. This work not only proposes novel ideas for the preparation of nano cobalt/carbon catalysts, but also facilitates the recycling and high-value utilization of waste plastics.

## Data Availability

Data are contained within the article and Supplementary Materials.

## Supplementary Materials

The following supporting information can be downloaded at: https://www.mdpi.com/article/10.3390/nano15050371/s1, Text S1: Chemicals; Text S2: Characterizations; Text S3: Catalytic degradation test; Table S1: Surface area and pore structure of PET-800 and CoPC catalysts; Table S2: Comparison of CoPC-2 catalyst with others Co-based catalyst; Table S3: Effects of catalyst dosage on TC degradation, fitted coefficient of determination, and rate constants; Table S4: Effects of PMS dosage on TC degradation, fitted coefficient of determination, and reaction rate constants; Table S5: Effects of initial TC concentration on TC degradation, fitted coefficient of determination, and reaction rate constants; Table S6: Effects of solution pH on TC degradation, fitted coefficient of determination, and reaction rate constants; Table S7: Effects of solution temperature on TC degradation, fitted coefficient of determination, and reaction rate constants; Figure S1: Adsorption effect of PET-800 and CoPC catalysts; Figure S2: Linear fitting of Arrhenius equation; Figure S3: Effect of N_2_ on TC degradation.
