# Supplementary material for: Nano Cobalt-Loaded Porous Carbon Derived from Waste Plastic for Efficient Persulfate Activation and Tetracycline Degradation
Source: Nanomaterials (Basel). 2025 Feb 27;15(5):371. doi: 10.3390/nano15050371 (PMC11901869; doi:10.3390/nano15050371)
Supplement: Supplementary file 1 [file nanomaterials-15-00371-s001.zip › nanomaterials-3421448-supplementary.pdf]

Nano Cobalt-Loaded Porous Carbon Derived from Waste Plastic for Efficient Persulfate Activation and Tetracycline Degradation

Yueyue Luo [1,†], Xiuxiu Zhang [1,†], Yu Zhang [1], Jianchao Wang [2,*] and Chongqing Wang [1,*]

1 Zhongyuan Critical Metal Laboratory, School of Chemical Engineering, Zhengzhou University, Zhengzhou 450001, China;

2 State Key Laboratory of Nutrient Use and Management, College of Resources and Environmental Sciences, National Academy of Agriculture Green Development, Key Laboratory of Plant–Soil Interactions (Ministry of Education), China Agricultural University, Beijing 100193, China

* Correspondence: jcwang91@163.com (J.W.); cqwang1990@zzu.edu.cn (C.W.)

† These authors contributed equally to this work.

Text S1. Chemicals

Text S2. Characterizations

Text S3. Catalytic degradation test

Table S1. Surface area and pore structure of PET-800 and CoPC catalysts.

Table S2. Comparison of CoPC-2 catalyst with others Co-based catalyst.

Table S3. Effects of catalyst dosage on TC degradation, fitted coefficient of determination, and rate constants.

Table S4. Effects of PMS dosage on TC degradation, fitted coefficient of determination, and reaction rate constants.

Table S5. Effects of initial TC concentration on TC degradation, fitted coefficient of determination, and reaction rate constants.

Table S6. Effects of solution pH on TC degradation, fitted coefficient of determination, and reaction rate constants.

Table S7. Effects of solution temperature on TC degradation, fitted coefficient of determination, and reaction rate constants.

Figure S1. Adsorption effect of PET-800 and CoPC catalysts.

Figure S2. Linear fitting of Arrhenius equation.

Figure S3. Effect of N_2 on TC degradation.

Text S1. Chemicals

Waste plastic samples were obtained from waste beverage bottles produced by COFCO Coca-Cola Beverages Co limited in Henan, China, whose main component was Polyethylene terephthalate (PET). TC, Cobalt acetate tetrahydrate $(CH_3COO)_2Co·4H_2O$, Trifluoroacetic acid (CF_3COOF), Ethanol (C_2H_6O), L-histidine ($C_6H_9N_3O_2$), isopropanol (C_3H_8O), furfuryl alcohol ($C_5H_6O_2$) and other medicines used in the experiment were purchased from Shanghai Rohn Chemical Technology Co located at Shanghai, China.

Text S2. Characterizations

To investigate the crystal structure of the different catalysts, an x-ray diffractometer (XRD) from PANalytical, Netherlands, was used in the range of 5-90° with a scanning speed of 10°/min. A Fourier Transform Infrared Spectroscopy (FT-IR), Nicolet iS10 from Thermo, USA, was used to characterize the functional groups on the catalyst surface, with a scanning range of 4000~400 cm^{-1} and a resolution of 4 cm^{-1}. The microscopic morphology of the catalysts was tested using a Gemini SEM 300 Field Emission Scanning Electron Microscope (SEM) from Zeiss, Germany, while the elemental compositions and contents were detected using an energy-dispersive X-ray spectrometer (EDS). The applied accelerating voltage was 5.0 kV. To verify the effect of different Co loadings on the degree of graphitization of the catalysts, the samples were tested by Raman spectroscopy using a Horiba LabRAM HR Evolution Raman spectrometer (Raman) with a laser wavelength of 532 nm. The specific surface area and pore structure of the catalysts were characterized by testing the N_2 adsorption-desorption curves of the samples using a Bayer BELSORP-mini fully automated specific surface and porosity analyzer (BET), Japan. The relative pressure P/P_0 of the test was between 0.05 and 1.0, and the test temperature was liquid nitrogen temperature (196 °C). To verify the presence of radicals in the CoPC/PMS system, Electron paramagnetic resonance spectroscopy (EPR) tests were carried out using Bruker EMX plus, Germany, with the unsaturated antimagnetic compounds 5,5-dimethyl-1-pyrroline-N-oxide (DMPO, 20 mM), and 2,2,6,6-tetramethylpiperidine (TEMP, 20 mM) as spin trapping agents. During the test, CoPC catalyst and PMS were dosed at 20 mg

and 1.1 mM, respectively. The test time was 0, 5, and 10 min, respectively.

Text S3. Catalytic degradation test

All catalytic degradation experiments were performed in a thermostatic oscillator. The thermostatic oscillator used was model WHY-2 manufactured by Jiangsu Jiamei Instrument Manufacturing Co, China. During the degradation process, the rotation speed was 200 r/min, and the initial temperature was 25 °C. For the experiment, the catalyst was added to the TC solution and shaken at constant temperature for 30 min to reach adsorption saturation, and then PMS for degradation reaction. A 2.5 mL sample of the TC solution was removed at time intervals, and the catalyst powder was filtered off using a 0.45 μm vacuum filter. The concentration of residual TC in the solution was immediately analyzed using a UV spectrophotometer (Lambda 850+, USA) at 354 nm. The TC degradation rate was calculated from the absorbance of TC at 354 nm.

Table S1. Surface area and pore structure of PET-800 and CoPC catalysts.

Catalysts	BET surface area (m^2/g)	Average pore diameter (nm)	Total pore volume of pores (cm^3/g)
PET-800	438.64	2.185	0.2397
CoPC-1	262.45	3.561	0.2337
CoPC-2	343.41	3.467	0.2976
CoPC-3	266.52	3.615	0.2409

Table S2. Comparison of CoPC-2 catalyst with others Co-based catalyst.

Catalysts	Pollutants	Experimental conditions	Removal efficiency	Reference
Co-AC	CIP	Catalyst: 0.75 g/L<br>PMS: 2 mM<br>CIP: 50 μM, 200 mL	100%<br>(120 min)	[7]
OMC-Co-T800	SMX	Catalyst: 0.1 g/L<br>PMS: 0.4 g/L<br>SMX: 10 mg/L, 50 mL	99%<br>(30 min)	[2]
Co/CNF	TC	Catalyst: 0.3 g/L<br>PDS: 1.0 g/L<br>TC: 20 mg/L, 100 mL	89.5%<br>(60 min)	[57]
Co-BC	NOR	Catalyst: 1.0 g/L<br>PS: 10 mM<br>NOR:20 mg/L, 100 mL	97.66%<br>(180 min)	[9]
FCOCN	TC	Catalyst: 0.6 g/L<br>PS: 2.5 g/L<br>TC: 15 mg/L, 100 mL	90.1%<br>(120 min)	[1]
Co-N-BC	OFX	Catalyst: 2.0 g/L<br>PS: 2.0 mM<br>OFX: 10 mg/L, 100 mL	93.99%<br>(120 min)	[8]
Co@RBC800	LVF	Catalyst: 0.2 g/L<br>PMS: 0.5 mM<br>LVF: 10 mg/L, 50 mL	100%<br>(15 min)	[32]
Co-GMC-900	CIP	Catalyst: 0.1 g/L<br>PMS: 0.4 g/L<br>CIP: 20 mg/L, 100 mL	96.5%<br>(30 min)	[48]
rGO-8CoPcS-SA	SMX	Catalyst: 0.086 g/L<br>PMS: 0.8 mM	100%<br>(45 min)	[5]

		SMX: 10 mg/L, 50 mL		
CoPC-2	TC	Catalyst: 0.2 g/L<br>PMS: 1.1 mM<br>TC: 20 mg/L, 200 mL	87.93%<br>(60 min)	This work

Table S3. Effects of catalyst dosage on TC degradation, fitted coefficient of determination, and rate constants.

Catalysts dosage (mg/L)	Removal rate (%)	R^2	k_{obs} (min^{-1})
20	83.077	0.9932	0.0521
40	85.300	0.9406	0.0852
60	88.091	0.8879	0.1145
80	88.808	0.8804	0.1200

Table S4. Effects of PMS dosage on TC degradation, fitted coefficient of determination, and reaction rate constants.

PMS dosage (mM)	Removal rate (%)	R^2	k_{obs} (min^{-1})
0	8.351	0.7026	0.0039
0.2	45.251	0.9120	0.0226
0.4	60.283	0.9178	0.0348
0.7	76.812	0.8925	0.0511
1.1	87.930	0.9618	0.0668
1.5	90.747	0.9632	0.0747

Table S5. Effects of initial TC concentration on TC degradation, fitted coefficient of determination, and reaction rate constants.

TC concentration (mg/L)	Removal rate (%)	R^2	k_{obs} (min^{-1})
10	99.094	0.9741	0.5202
30	91.452	0.9501	0.1699
50	87.930	0.9780	0.1374
70	66.280	0.9428	0.0806

Table S6. Effects of solution pH on TC degradation, fitted coefficient of determination, and rate constants.

pH	Removal rate (%)	R^2	k_{obs} (min^{-1})
2	52.421	0.9899	0.0169
4	87.930	0.9618	0.0668
6	87.475	0.9544	0.0698
8	87.620	0.9145	0.0761
10	87.906	0.9195	0.0774
12	60.393	0.9823	0.0183

Table S7. Effects of solution temperature on TC degradation, fitted coefficient of determination, and rate constants.

Temperature (°C)	Removal rate (%)	R^2	k_{obs} (min^{-1})
25	87.930	0.9703	0.0802
30	88.777	0.9381	0.1048
35	88.986	0.9226	0.1112
40	89.527	0.8745	0.1321

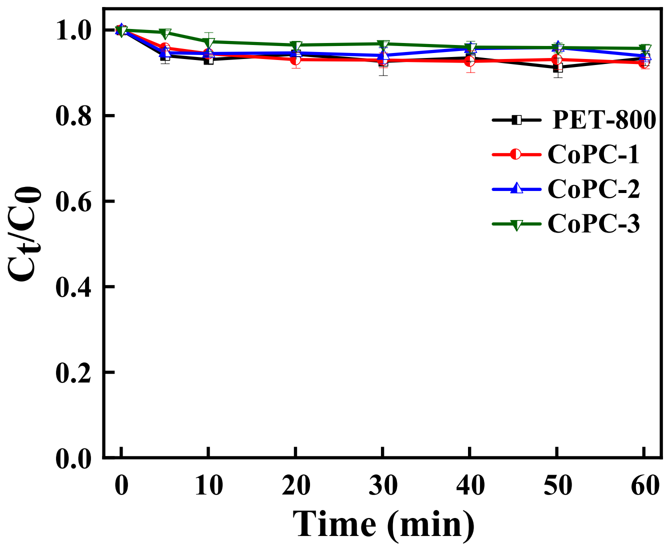

Figure S1. Adsorption effect of PET-800 and CoPC catalysts.

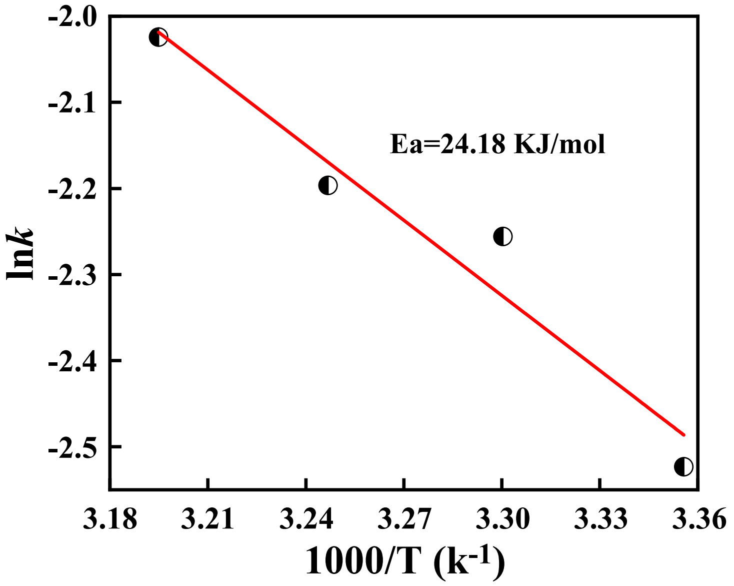

Figure S2. Linear fitting of Arrhenius equation.

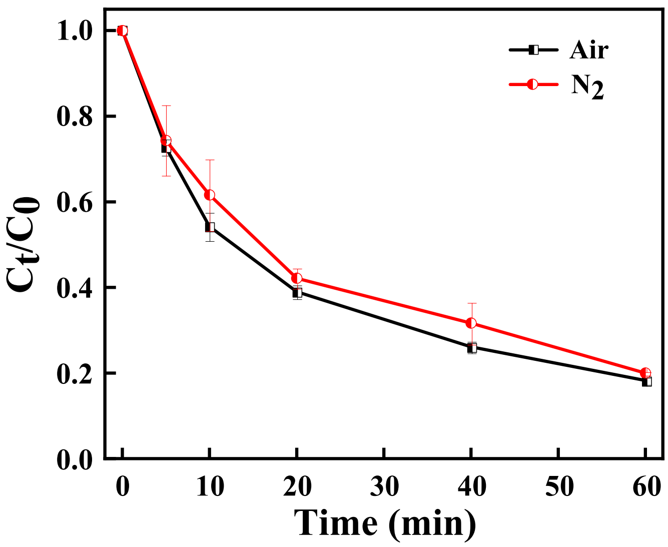

Figure S3. Effect of N_2 on TC degradation.
